# Factors associated with severity and anatomical distribution of diabetic foot ulcer in a tertiary care hospital in Bangladesh: A cross-sectional study

**DOI:** 10.1097/MD.0000000000040510

**Published:** 2024-11-15

**Authors:** Md. Abdus Salam, Md. Raihan Kabir Ziko, Arefin Naher Oishee, Anuj Yadav, Md. Abdul Monaem, Abdullah Salman, Suman Kadariya, Faisal Chowdhury, Shashwat Kafley, Mushfiqur Rahman Pulok, Unika Kc, Rimsa Subedi, Aman Babu Shrestha

**Affiliations:** a Department of Surgery, M Abdur Rahim Medical College Hospital, Dinajpur, Bangladesh; b M Abdur Rahim Medical College, Dinajpur, Bangladesh; c Colonel Malek Medical College, Manikganj, Bangladesh; d KIST Medical College and Teaching Hospital, Imadol, Lalitpur, Nepal; e Chittagong Medical College, Chittagong, Bangladesh; f Enam Medical College, Dhaka, Bangladesh; g Gandaki Medical College, Pokhara, Nepal; h Kathmandu University School of Medical Sciences, Dhulikhel, Nepal; i Islington College, Kathmandu, Nepal.

**Keywords:** anatomical distribution, diabetic foot ulcer, emotional well-being, general health

## Abstract

Diabetic foot ulcers are characterized by disturbances in the epidermis and/or a portion of the dermis in patients with the disease. With over a million amputations performed annually, it has also emerged as one of the primary causes of lower limb amputation globally. To better understand the severity and anatomical distribution of ulcerated areas in patients with type II diabetes mellitus, this study aimed to identify the factors associated with diabetic foot ulcers (DFUs). This descriptive cross-sectional study was conducted at M Abdur Rahim Medical College Hospital in Dinajpur, Bangladesh, from July to September 2023. The study population was selected using a purposive sampling technique based on the patients’ availability during their usual and regular treatment at MARMCH. Using a Bangla questionnaire data was obtained to evaluate the DFUs, in addition to measuring blood pressure and assessing the affected area’s neurological function. The severity of the ulcer is calculated by using the Wagner grading system. Data was analyzed by using STATS v15 and chi-square was applied. A total of 113 DFU patients took part in this study. The mean age in years was 56 ± 12 (SD + mean) and the male proportion was greater (61.9%). Most of them (93.91%) were negligent about foot care and suffered from severe DFU (86.37%). The majority of respondents (57.94%) had a right foot ulcer, of which 94.50% had severe ulcers. Almost all ulcers were severe (86.14%) and measured <5 cm in diameter (69.71%). The results highlight the tremendous burden of DFUs, which can have serious consequences and substantial mental and economic effects on patients’ healthcare systems.

## 1. Introduction

Diabetes mellitus is a chronic metabolic disorder characterized by elevated blood glucose levels which may occur due to a deficiency or resistance to the hormone, insulin.^[1]^ The prevalence of diabetes worldwide was estimated to be 9.3% in 2019 which is expected to rise to 10.2% by 2030.^[2]^

Bangladesh is a developing country located in South Asia, bordering India and Myanmar and according to the International Center for Diarrhoeal Disease and Research in Bangladesh (icddr, b) an estimated 13.1 million cases of diabetes mellitus were present in Bangladesh in 2021 AD with an estimated 75,617 deaths attributed to it in the same year.^[3]^ One of the advanced complications of diabetes mellitus is diabetic foot ulcer (DFU) with diabetic patients having about 15% chance of developing DFU throughout their lifetime, globally.^[4]^

A DFU is defined as a disruption in the epidermis and at least a part of dermis in a patient with diabetes. Certain lesions that do not penetrate up to the dermis are classified as pre-ulcerative lesions but have an increased chance of progression to ulcer.^[5]^ Diabetic foot ulcers can be classified into 3 categories namely neuropathic, ischemic, and neuro-ischemic with a prevalence of 35%, 15%, and 50%, respectively.^[6]^

Diabetic foot ulcers have also become one of the leading causes of lower limb amputation worldwide with over 1 million amputations done every year.^[7]^ The majority of all diabetes-related amputations are usually preceded by foot ulcers.^[8]^ It has also been shown that the occurrence of DFU has been associated with factors such as age, increased duration of diabetes mellitus, hypertension, microvascular complications such as diabetic retinopathy and a history of smoking.^[9]^ In addition to this other factors such as low educational status, high body mass index (BMI) and poor self-care of the foot also play a role in the progression of DFUs.^[10–12]^

During the care and treatment of DFUs, it has been shown that the involvement of a multidisciplinary team can play a significant role in reducing the risk of adverse outcomes and complications including reduction in amputation rates, lowering the total expenses endured by the patient and allowing a better quality of life for patients living with DFUs. A multidisciplinary approach to the care and treatment of DFUs has been associated with a decrease in the risk of amputation by 49% to 85%.^[13]^ An observational study conducted between March 2016 and April 2017 in 6 different healthcare institutions in Nigeria identified the major predictors of DFUs which included factors such as duration of ulcer for >1 month, Wagner grade 4 or above, leukocytosis, proteinuria, osteomyelitis, and infection of the wound.^[14]^

To determine the factors related to DFUs with the severity and anatomical distribution of the ulcerated area of the patients suffering from type II diabetes mellitus, we performed this study to evaluate the severity of this disease burden.

## 2. Materials and method

### 2.1. Design and location of the study

This descriptive cross-sectional research was conducted at M Abdur Rahim Medical College Hospital located in Dinajpur, Bangladesh, from July to September 2023.

### 2.2. Selection and recruitment of patients

The inclusion criteria encompassed individuals 18 years and older who had received a diagnosis of type 2 diabetes mellitus. The patients presented with a below ankle lesion and were undergoing treatment inside the department of surgery of M Abdur Rahim Medical College Hospital. The exclusion criteria encompassed individuals who were younger than 18 years old, as well as those who had cognitive impairments that rendered them incapable of communication and providing consent. Patients who did not possess adequate mental capacities to understand and provide consent were also excluded from participation in this study. The selection of participants was conducted using the purposive sampling procedure. After explaining the research protocol, patients were requested to provide written informed consent to participate in this study.

### 2.3. Data collection procedure

Calculation of the sample size was performed utilizing the Kish-Leslie formula^[15]^ Given the absence of sufficient empirical research on the prevalence of DFUs among the Bangladeshi population, an estimation of prevalence was derived from cross-sectional research on DFU conducted in India, which reported a prevalence rate of 4.5%.^[16]^

The Kish Leslie formula:


$$\begin{array}{l} n=\frac{z^{2}pq}{d^{2}} \end{array}$$

Here,

n = the desired sample size.

*z* = the value of standard variance at a given confidence level (1.96 at 95% confidence interval).

*p* = incidence of DFU in patients suffering from DM 4.5% (0.045) (Sinharay et al, 2012).

*q* = 1‐*P* = 1‐0.045 = 0.955.

d = desired level of precision, which is considered as 0.05.

So,


$$\begin{array}{l} n=\frac{{\left(1.96\right)}^{2}\times 0.045\times 0.955}{{\left(0.05\right)}^{2}} \end{array}$$

$$\begin{array}{l} =\frac{0.16250}{0.0025}=66.03≅67 \end{array}$$

The data collection questionnaire was initially developed and piloted with the assistance of relevant scholarly sources. The questionnaire was translated into Bangla by a skilled linguist. Following this, the physicians who had undergone specialized training proceeded to carry out a thorough physical examination of the specified individuals. The methodology encompassed the assessment of the DFU, alongside the examination of the neurological condition of the affected region and the measurement of blood pressure. The physician assessed the severity of the ulcer using the Wagner grading system. The ankle-brachial index was calculated by utilizing the ratio of blood pressure measurements taken at the ankle of the affected foot and the distal third of the arm. Patients with ankle involvement due to DFU were omitted from the measurement of the ankle-brachial index, and this particular variable was designated as “non-applicable.”

Socio-demographic information, generic characteristics of the participants, and behavior-related variables were incorporated into the questionnaire. The sociodemographic data encompassed variables such as age, gender, marital status, occupation, place of living, and income per month. General characteristics of DM, including type, duration, received therapy, and type of therapy, are taken into account. Diet, past smoking history, alcohol use, follow-up consultation frequency, foot care, and overall lifestyle were all considered behavior-related factors. The dietary patterns were additionally classified as follows: carbohydrates (including rice, potatoes, sweet pumpkin, bread, and crackers), fatty foods (including nuts, ghee, walnuts, cheese, and oil), and fruits, vegetables, and nonvegetarians (including fish, meat, eggs, legumes, milk, and pulses).

The dietary consumption patterns were split into 4 distinct groups according to the frequency of consumption. The frequency categories for consumption were daily (patients consumed the indicated foods on a daily basis), usually (patients consumed the indicated food at a minimum frequency of 4 to 6 times per week), sometimes (patients consumed the indicated food at a frequency of 2 to 3 times per week), and seldom (patients consumed the indicated food once per week or less frequently).

Individuals who engage in frequent alcohol consumption, regardless of the specific type of alcohol ingested, were classified as alcoholic. On the other hand, individuals who consume alcohol only sometimes or have abstained from alcohol for a minimum of 2 years were classified as nonalcoholic. Patients were classified as smokers if they engaged in daily use of tobacco products, regardless of their form. A patient who never used tobacco products or who stopped using them for at least 2 years was considered a nonsmoker.

The patients’ frequency of diabetes consultation was classified according to their frequency of checkups at the diabetic clinic. The categories encompassed in the study were semiannual, annual, and never visited. The evaluation of the patient’s foot care practices entailed collecting data about their chosen implement for nail trimming (nail cutter or razorblade), the person accountable for performing the nail trimming (self or assisted), and their consistent footwear selection (closed, open, or fitted). The evaluation of foot care was performed utilizing a foot score, which entailed the allocation of points for certain acts. A single point was allocated for the act of cleansing the feet, while 2 points were assigned for the action of drying the interdigital spaces. Additionally, 3 points were granted for the utilization of moisturizing goods. A higher score was indicative of the patient’s engagement in all 3 tasks, while a moderate score suggested that the patient engaged in any 2 tasks, and a low score indicated that the patient only engaged in one of the 3 tasks.

The weight of each participant was measured with precision to the closest 100 g using a digital weight scale (model EB9013, Camry, Hong Kong). The measurement of height was conducted using a manual stadiometer equipped with a marker at the 200cm level. Subsequently, the recorded measurements of height and weight were employed to calculate the BMI. The BMI was calculated using the formula BMI = kg/m^2^, where weight is measured in kilograms and height is measured in meters. The standard classification of BMI by the World Health Organization categorizes individuals into 4 distinct groups based on their BMI values: individuals below 18.5 kg/m^2^ as underweight, individuals between 18.5 and 24.9 kg/m^2^ as normal, individuals between 25 and 29.9 kg/m^2^ as overweight, and individuals at and above 30 kg/m^2^ as obese.^[17]^ We used a digital sphygmomanometer (HEM-7156T, Omron, Japan), equipped with a cuff of suitable size for the patient’s arm to measure the blood pressure of the participants. The definition of hypertension employed in this study was derived from the criteria provided by the European Society of Cardiology. According to these criteria, hypertension is defined as having a systolic blood pressure of 140 mm Hg or higher, or a diastolic blood pressure of 90 mm Hg or higher.^[18]^

The physical examination was utilized to evaluate the attributes of the foot ulcers on the patients, encompassing the quantity, dimensions, and site of the ulcers. These factors were subsequently categorized according to the Wagner classification.^[19]^ The patients were categorized into 2 groups within the system: early DFUs, comprising grade 1 and grade 2 ulcers, and severe or late ulcers, encompassing grade 3 and above.^[20]^

The assessment of pressure sensation involved the utilization of a Semmes Weinstein monofilament 5.07/10 g (Manufactured in China). The monofilament was administered to the plantar surface of the foot, primarily targeting the base of the hallux, the second and fifth digits, as well as the calcaneus. The researchers made a deliberate effort to steer clear of the regions exhibiting callosity.^[21]^

The vibration sensation was assessed by employing a 128 Hz tuning fork on the hallux.^[21]^ The classification system employed in this study for diagnosing diabetic neuropathy was based on Picon et al’s recent classification, which includes 4 categories: nonexistent, minor, moderate, and severe.^[22]^

For assessing the severity of diabetic peripheral neuropathy among participants, we applied the neuropathy disability score. The tool assesses the degree of peripheral neuropathy on a scale of 0 to 10 based on 4 parameters. The parameters encompassed in this study involve the assessment of various sensory sensations in the lower extremities. These sensations include the perception of vibration induced by a 128 Hz tuning fork, with a binary scale of 0 indicating the presence and 1 indicating a reduction or absence of sensation for each foot. Similarly, the perception of temperature induced by a cold tuning fork is evaluated using the same binary scale. Additionally, the pin-prick sensation is assessed using a monofilament, with a binary scale of 0 indicating the presence and 1 indicating a reduction or absence of sensation for each foot and ankle reflex. Lastly, the Achilles tendon reflex is evaluated using a patellar hammer, with a scale of 0 indicating normal reflex, 1 indicating presence with reinforcement, and 2 indicating absence per side.^[23]^ An absence of neuropathy (normal) was defined as a score ranging from 0 to 2. In addition to the aforementioned classifications, impairment resulting from diabetic peripheral neuropathy can be further categorized. The levels of intensity can be classified into 3 categories: mild, moderate, and severe. Mild intensity falls within the range of 3 to 5, moderate intensity falls within the range of 6 to 8, and severe intensity falls within the range of 9 to 10.^[24]^

### 2.4. Sample collection and biochemical analysis

Following disinfection with 70% alcohol, 10 mL of venous blood was obtained from the superficial veins of the cubital fossa using a sterile disposable plastic syringe. The sample was collected for biochemical analysis, which included the patient’s fasting blood sugar (FBS), HbA1c, fasting lipid profile, complete blood count, and serum creatinine. The blood samples were placed in a 4-liter isolated refrigerated medical box (Manufactured in China) and promptly transferred to the laboratory for analysis. The measurement of a whole blood count was conducted utilizing a hematology analyzer (CTS-3000, Celltus, Barcelona, Spain). The samples were maintained at ambient temperature in order to induce coagulation, after which they were subjected to centrifugation at a speed of 4400 revolutions per minute for 10 minutes using (Sorvall ST 8R, Thermo Scientific, Bremen, Germany). The extracted serums were maintained in a refrigerator at a temperature of −20 °C and then analyzed for the presence of biochemical markers. Serum levels of FBS, HbA1c, total cholesterol, both high and low-density lipoprotein cholesterol, and triglyceride and serum creatinine were assessed using colorimetric methods on a biochemistry analyzer (Humalyzer 3000, USA) with readily accessible diagnostic kits (Human Diagnostic, Germany).^[25]^

Optimal biochemical parameters were defined as FBS levels <7.0 mmol/L, HbA1c levels below 7%, low-density lipoprotein cholesterol levels below 100 mg/dL, high density lipoprotein levels above 40 mg/dL for males and above 50 mg/dL for females, triglyceride levels below 150 mg/dL, and serum creatinine levels ranging from 0.8 to 1.4 for males and 0.6 to 1.2 for females.^[26]^

### 2.5. Data processing and analysis

The messy data was entered into Microsoft Excel. After sorting, the data was transmitted to Stata version 15 (Stata Corp®) for analysis. Proportions were employed for the study of categorical variables, and the chi-square test was utilized to determine any significant differences in proportions. The data was presented in the format of frequencies and percentages. The researchers utilized means and standard errors to depict the continuous variables.

### 2.6. Ethical permission

Ethical permission was obtained from the IRB committee of M Abdur Rahim Medical College Hospital, Dinajpur.

## 3. Result

A total of 113 patients with DFU participated in this study. The mean age in years was 56 ± 12.64 (SD ± mean). Participants between 40 to 49 years had the highest incidence of DFU (27.08%, n = 26). Males (61.9%, n = 70) and patients from the village (85.29%, n = 81) had a higher prevalence of developing severe DFU as depicted in Table 1.

**Table 1:** Socio-demographic status of patients with DFU.

Variables	Less severe DFU (G1 & G2): n (%)	Severe DFU (G3, G4, & G5): n (%)	Total n (%)	*P*-value
Age group in years				.339
18–39	6 (35.29)	10 (10.42)	16 (9.34)	
40–49	1 (5.88)	26 (27.08)	27 (25.23)	
50–59	1 (5.88)	22 (22.92)	23 (21.49)	
60–69	3 (17.65)	22 (22.92)	25 (23.36)	
70–95	6 (35.29)	16 (16.67)	22 (20.56)	
Sex				.469
Male	14 (81.82)	56 (61.32)	70 (61.9)	
Female	3 (18.18)	40 (38.68)	43 (38.05)	
Salary				1.754
<16,000	9 (53.33)	9 (11.22)	19 (12.14)	
>16,000	7 (46.67)	87 (88.77)	94 (87.85)	
Residence				.391
City	0 (0)	15 (14.71)	15 (13.27)	
Village	17 (100)	81 (85.29)	98 (86.72)	
Occupation				.531
Farmer	6 (32.12)	31 (41.41)	39 (33.11)	
Business/self-employed	5 (24.24)	34 (44.57)	39 (33.16)	
Not employed	7 (43.63)	12 (13.51)	38 (34.01)	

DFU = diabetic foot ulcer.

Table 2 describes, the majority of the participants 93.91% (n = 86) with a score of 1 in foot care were extremely negligent regarding their feet, with 86.37% (n = 76) of them suffering from severe DFU. Despite suffering from severe DFU (62.80%, n = 79), the majority of patients (79.43%, n = 85) only visited the hospital annually. The prevalence of additional behavioral factors that contribute to severe DFU is also depicted.

**Table 2:** Behavioural attributes of patients suffering from DFU.

	Less severe (1, 2)	Severe (3, 4+)	Total (n%)	*P*-value
Hospital visits				.621
1 time a year	6 (42.10%)	79 (62.80%)	85 (79.43)	
1 time in 6 months	3 (15.78%)	13 (34.02%)	16 (14.95)	
Never went	8 (42.10%)	4 (3.17%)	12 (5.60)	
Nail cutter				.202
Yes	7 (37.50%)	58 (60.20%)	65 (57.94)	
No	10 (62.50%)	39 (39.80%)	49 (42.05)	
Razor blade				.829
Yes	10 (63.36%)	15 (67.47%)	29 (27.10)	
No	7 (37.64%)	10 (33.53%)	78 (72.89)	
Knife				.195
Yes	6 (37.50)	13 (13.42)	19 (14.95)	
No	10 (62.50)	84 (86.58)	94 (85.04)	
Foot care				.165
1	17 (100)	76 (88.37)	86 (93.91)	
2	0	3 (5.81)	3 (2.8)	
3	0	3 (5.81)	3 (2.8)	
Types of shoes worn				.385
Sandal	13 (90.91)	90 (90.29)	103 (96.26)	
Any type of shoes	4 (9.09)	4 (6.80)	8 (1.86)	
Do not wear shoes	0	2 (2.91)	2 (1.97)	
Individuals who cut their toenails				.732
Self	8 (53.85)	84 (87.63)	92 (83.177)	
Other	9 (46.15)	18 (12.37)	21 (16.822)	

DFU = diabetic foot ulcer.

The majority of respondents (57.94%, n = 65) had a right foot ulcer, of which 94.50% (n = 50) were severe. The majority of the ulcers were severe (86.14%, n = 88) and measured <5 cm in diameter (69.71%, n = 96). Nearly half of the respondents (46.21%; n = 55) had a single ulcer of which 58.82% (n = 51) were severe. Greater than 4 ulcers (*P* < .05) and an ulcer size >5 cm were both associated with severe DFU that as shown in Table 3.

**Table 3:** Anatomical distribution of DFU among patients.

	Less severe	Severe	Total (n%)	*P*-value
Foot affected by DFU				.531
Right	15 (46.02)	50 (50.94)	65 (57.94)	
Left	8 (25.23)	15 (33.54)	23 (20.54)	
Both	9 (28.74)	16 (14.51)	25 (23.51)	
Size of DFU in cm				.004
1–5	8 (41.81)	88 (86.14)	96 (69.71)	
5–10	9 (59.13)	12 (13.91)	17 (30.28)	
No of ulcer				.031
1	3 (40.32)	51 (58.82)	55 (46.21)	
2	3 (27.42)	26 (23.53)	29 (24.37)	
3	7 (24.19)	13 (9.80)	20 (16.81)	
4	4 (24.19)	15 (7.84)	19 (15.96)	

DFU = diabetic foot ulcer.

## 4. Discussion

Diabetic foot ulcer (DFU) stands as one of the most frequent, severe, and costly complications arising from diabetes, and it represents a primary reason for hospital admissions globally.^[27]^ Treating DFUs incurs substantial financial burdens, with expenditures nearing $11 billion in the United States and $456 million in the United Kingdom.^[28]^ The prevalence of DFUs among diverse populations ranges from 3% to 18%.^[29]^ DFUs frequently lead to elevated amputation rates, emotional distress, socioeconomic challenges, diminished quality of life, and, in some cases, even mortality.^[30]^ This research assesses the anatomical distribution of DFUs and explores the factors associated with their severity among patients with diabetes mellitus at a tertiary care hospital in Bangladesh.

The burden of DM and DFU is increasing worldwide^[31]^ Adult patients with diabetes face a notable 10% to 15% risk of developing a DFU during their diabetic lifetime.^[32]^ As per our study, patients of age between 40 to 49 years had the highest incidence of DFU (27.08%, n = 26) which was statistically insignificant to conclude that increasing age is associated with higher chances of DFU. In contrast, a study conducted by Jeyaraman et al^[33]^ reported a correlation between age (with a median age of 64 years) and the occurrence of DFUs. This study suggested that older age was associated with a heightened risk of angiopathy, and individuals aged over 40 years appeared to be more susceptible to developing this condition.^[34]^ Jeffcoate et al^[35]^ also observed that older individuals encountered greater challenges in wound healing when dealing with DFUs. This phenomenon may be attributed to the deterioration of vascular function with advancing age, resulting in a higher infection rate in the elderly compared to younger age groups.^[36]^

Although our study had a male majority, sex was not significantly associated with the severity of DFU. The prevalence of males in our study aligns with previous research, which has also shown a male preponderance in DFU cases.^[37]^ However, a worldwide systematic review and meta-analysis by Jupiter et.al found that male patients were notably more prone to severe DFU compared to their female counterparts.

In our study, a majority of patients presented with severe DFU (n = 96, 84.9%), a finding consistent with a study conducted by Smith-Strom in Norway.^[38]^ Nevertheless, opposing results have been documented by Jalilian and colleagues in a systematic review, where a higher proportion of patients exhibited less severe DFU.^[20]^ In light of these findings, it becomes evident that the development of clear guidelines for the prevention and treatment of DFU, tailored to our specific context and based on robust evidence, could play a pivotal role in alleviating the burden of complications stemming from DFU. The majority of patients in our study exhibited Wagner classification Grade 3 DFUs, a finding that aligns with the results of a study conducted in Sri Lanka.^[39]^ In contrast, research from Ethiopia and India has reported that Wagner classification Grade 2 DFU is the most prevalent among diabetic patients.^[40–42]^ Despite the high prevalence of severe DFU in our study (62.80%, n = 79), it is noteworthy that the majority of patients (79.43%, n = 85) sought medical attention only on an annual basis. This underscores the importance of patient education in the context of diabetes management, emphasizing the need for early medical consultation in the event of any foot injury to reduce the likelihood of DFU progression and its associated complications in our setting.

Living in a rural area with a diabetes mellitus diagnosis was associated with a higher incidence of DFU (n = 98, 86.72%) compared to urban areas, a correlation that has been reported in other studies as well, linking these factors to the development of severe DFU.^[10]^ Additionally, our findings revealed that DFU was predominantly located on the right foot, a result consistent with the observations of Smith-Strøm in Norway.^[38]^ Moreover, most patients in our study presented with DFUs measuring <5 cm^2^, a finding akin to a study conducted in Uganda where DFUs measuring <5 cm^2^ were the most commonly observed.^[43]^

The presence of more than 4 ulcers (*P* < .05) and an ulcer size exceeding 5 cm were both found to be linked with the development of severe DFUs. This study highlighted a clear trend: larger ulcers were more likely to progress to a severe state. As a result, it is crucial to commence early and effective treatment for DFUs in order to prevent their progression toward severity. Notably, Ambageda study in Sri Lanka did not identify any significant associations between various factors and the severity of DFU.^[39]^ Numerous studies have investigated factors associated with the severity of DFUs in diabetic patients, revealing diverse findings.^[44,45]^ For instance, a study conducted in Norway identified the duration of the ulcer as a factor associated with its severity, while a systematic review by Campbell reported a link between high HbA1c levels and increased DFU severity.^[44,45]^ Additionally, a systematic review by Jalalian noted that the location of the DFU on the plantar region was associated with a higher likelihood of severe DFU.^[20]^

Given the apparent variability in factors influencing the severity of DFUs across different regions, it is advisable to conduct location-specific research to tailor strategies for mitigating DFU complications effectively.

## 5. Conclusion

In conclusion, this study sheds light on the prevalence and factors associated with the severity of DFUs among patients with diabetes mellitus in Bangladesh. The findings emphasize the significant burden of DFUs, which can lead to severe complications and impose substantial financial and emotional costs on individuals and healthcare systems. Notably, a considerable proportion of patients sought medical attention only annually, highlighting the importance of patient education and early intervention to prevent the progression of DFUs. These findings underscore the need for targeted interventions and preventive strategies tailored to the specific context of Bangladesh. A multidisciplinary approach, including patient education, regular foot care, and early medical consultation, is crucial for reducing the risk of adverse outcomes associated with DFUs. Numerous lifestyle and self-care factors, as well as health system measures, may be useful in preventing serious pressure ulcers. Additionally, the study emphasizes the importance of understanding regional variations in factors influencing DFU severity to implement effective mitigation strategies. Providing teaching and training for care-providers of patients due to knowing factors and reducing them, proper hygiene and foot-care teaching and training for patients to reduce the risk factors are the recommendation for reducing this disease. Addressing the challenges posed by DFUs requires a comprehensive and context-specific approach, involving healthcare providers, policymakers, and the community. Further research and continuous monitoring are essential to refine strategies and improve the management of DFUs in Bangladesh and similar settings worldwide.

## Author contributions

**Conceptualization:** Md. Abdus Salam, Md. Raihan Kabir Ziko.

**Data curation:** Arefin Naher Oishee, Anuj Yadav, Md. Abdul Monaem, Suman Kadariya, Mushfiqur Rahman Pulok.

**Formal analysis:** Anuj Yadav, Shashwat Kafley, Rimsa Subedi, Aman Babu Shrestha.

**Methodology:** Md. Abdus Salam, Md. Raihan Kabir Ziko, Anuj Yadav, Abdullah Salman, Md. Abdul Monaem, Aman Babu Shrestha.

**Project administration:** Faisal Chowdhury, Shashwat Kafley, Unika Kc.

**Resources:** Suman Kadariya.

**Supervision:** Md. Abdus Salam, Md. Raihan Kabir Ziko.

**Validation:** Abdullah Salman, Faisal Chowdhury, Aman Babu Shrestha.

**Writing – review & editing:** Arefin Naher Oishee.

**Writing – original draft:** Abdullah Salman, Md. Abdul Monaem, Suman Kadariya, Faisal Chowdhury, Unika Kc, Rimsa Subedi.
